# Life-long coping patterns and bio-psycho-social predictors of individual responses to the COVID-19 pandemic—protocol of the Rostock Longitudinal Study (ninth wave)

**DOI:** 10.3389/fpubh.2026.1753885

**Published:** 2026-03-02

**Authors:** Elisa Wandinger, Antonia Brindle, Frauke Nees, Olaf Reis

**Affiliations:** 1 Department of Child and Adolescent Psychiatry and Neurology, University Medical Centre Rostock, Rostock, Germany; 2 German Center for Child and Adolescent Health, Site Greifswald/Rostock, Rostock, Germany; 3 Department of Child and Adolescent Psychiatry and Psychotherapy, Central Institute of Mental Health, Mannheim, Germany; 4 Institute of Medical Psychology and Medical Sociology, University Medical Centre Schleswig-Holstein, Kiel University, Kiel, Germany; 5 Institude of Medical Psychology, Ludwigs-Maximilians-University Munich, Munich, Germany

**Keywords:** coping with COVID-19, health behaviour, life-long trajectories, longitudinal study, public health

## Abstract

**Background:**

When faced with adversity and negative life events, such as infection with coronavirus or restrictive measures regarding COVID-19, individuals tend to rely on the same coping strategies they have already employed in the past. However, habitual coping measures vary greatly between people. Differences in dealing with challenging situations may depend on life-long coping experiences and a wide range of bio-psycho-social influences. The major goal of this study is to detect persisting patterns of coping which in turn may predict how individuals have handled the challenges of the COVID-19 pandemic. Using a prospective data set of 55 years we want to test the hypothesis of individual accentuation during crises by Caspi and Moffit.

**Methods:**

The Rostock Longitudinal Study (ROLS) is a prospective, epidemiological, observational study that started in 1970 with 300 new-borns in the north of Eastern Germany. Since then, a mixture of biological, social, and psychological factors has been collected in 1972, 1976, 1980, 1984, 1990, 1995, and 2008. The 9th wave measures a wide range of biological (physical health, neurobehavioural regulation, genetic analysis), psychological (personality facets, psychopathology, coping styles) and social (social network characteristics) risk and protective factors. The data will be analysed using growth curve modelling and prediction techniques. As part of the CoviDrug consortium, findings from the ROLS will be compared to cohorts of other longitudinal studies in Germany.

**Discussion:**

Changes to everyday life due to the COVID-19 pandemic have been unprecedented and extreme adaptability is required to maintain physical and mental health during this time of crisis. This study aims to investigate how individuals have coped with the challenges of the COVID-19 pandemic whilst taking into account individual and existing contextual factors in a bio-psycho-social model over the life course. It specifically looks into health behaviour as a potential outlet of coping with the widespread occurrence of the infectious SARS-CoV-2 virus.

**Clinical trial registration:**

https://drks.de/search/en/trial/DRKS00036469, identifier: DRKS00036469.

## Introduction

The widespread impact of the COVID-19 pandemic on both mental and physical health as well as the economic burden presented an unprecedented challenge to individuals and societies worldwide, highlighting the need for research into key coping mechanisms—such as which individuals were most or least affected, how they adapted to pandemic-related changes, and what personal and contextual factors influenced their adaptability. When individuals perceive that the demands from external situations are beyond their capacity, stress—a feeling of emotional strain or pressure—occurs (1). Coping can be described as ways of dealing with these stressful situations and/or emotions, including both volitional and automatized, cognitive, emotional, and behavioural responses to stress (2). Coping plays a critical role in the life-long development of resilience (3) and has strong associations with measures of mental health and well-being (4). Most taxonomies used in coping research distinguish between two basic categories: problem-focused and emotion-focused coping. These are strategies aimed at “managing or altering the problem causing the distress” and “regulating emotional responses to the problem,” respectively (1). Another commonly used distinction is that between engagement (i.e., taking charge) versus disengagement (i.e., diverting from the stressor) coping.

On one side, coping strategies depend on context and their effectiveness differs according to the situation (5). The famous freeze-flight-fight trichotomy (6) describes the simplified portfolio of behaviours in response to threat that are more (or less) applicable and effective in certain conditions. However, regardless of the characteristics of the stress-inducing situation, past research has reported a distinction between functional and dysfunctional strategies based on associations with psychopathology. Behavioural disengagement, self-blame, rumination, suppression, and substance use have been shown to be maladaptive (7) as they may have short time advantages in alleviating stress responses but detrimental long-term consequences such as increased risk for depression (8). Functional approaches, on the other hand, for example reappraisal or active problem-solving, have negative associations with psychopathology (9).

Individuals differ greatly in their responses to stressors. However, they are apt to show similar behavioural reactions over time across situations and therefore have a consistent way of responding to stress-inducing conditions. Moreover, individuals do not rely on a single coping strategy when faced with negative events, but rather have a set of preferred strategies at their disposal (10) that can be used simultaneously or successively to cope with stressors. Given that coping is a lifelong and learnable process, individuals tend to develop coping profiles that align with their inherent personal characteristics, such as gender, personality, neuropsychological functioning, and social support patterns. Over time, these factors contribute to the formation of a distinct coping trajectory, characterised by the progressive development of a personalised repertoire of habitual coping strategies.

### Factors influencing coping

**Figure 1:**
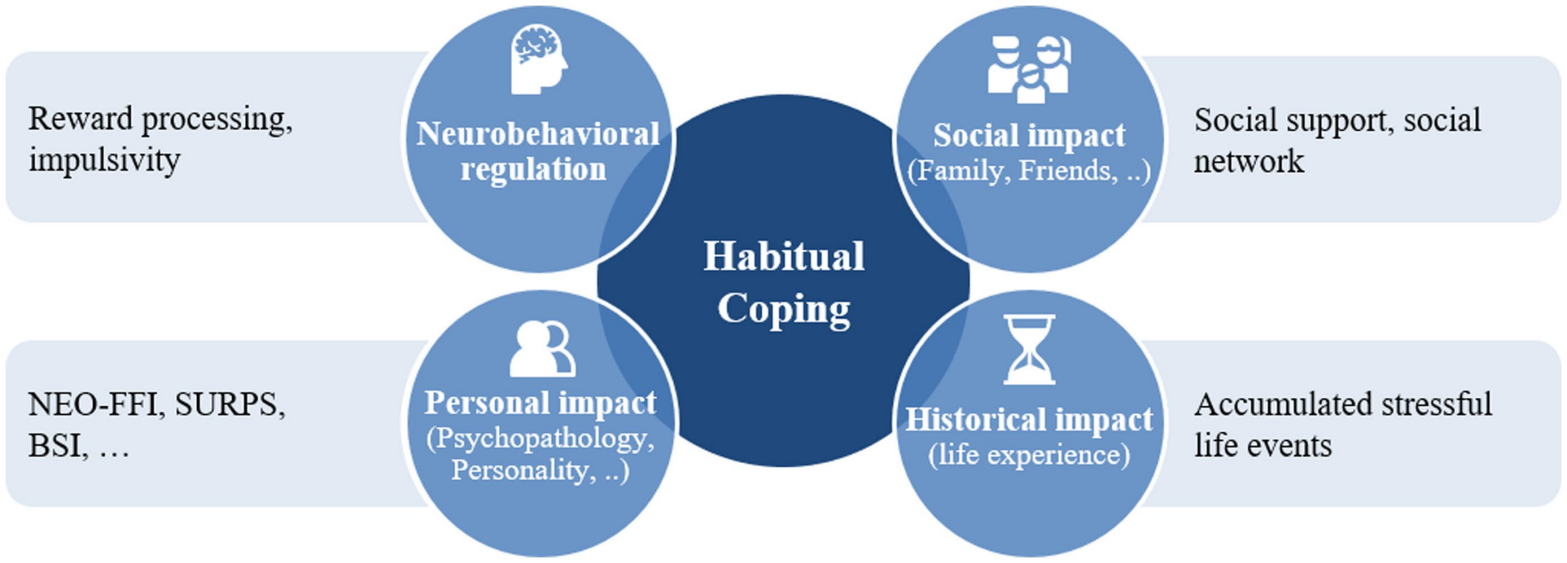
Bio-psycho-social impact factors of coping.

In a complex model, coping and its development over time depends on a wide array of influences, as it is shown in Figure 1. Bio-psycho-social models of health and resilience emanate from a holistic viewpoint and acknowledge the complex interplay of various factors influencing how individuals deal with stressful situations.

#### Neurobehavioural representation of coping

Past research has identified several executive functions as important mechanisms in coping (11). Inhibition, for instance, refers to the ability to selectively suppress prepotent or affectively driven behaviours and allows for a flexible response to situational changes. It is involved in the ability to select and apply adaptive strategies and is needed for planning or positive reframing of a stressful situation. Deficits in inhibition negatively affect coping ability and have been associated with depressive symptoms (12). Repeated and prolonged states of stress have been shown to deteriorate cognitive inhibition and enhance response inhibition, leading to a cognitive state of reactive and automatic processing (13). Activity in the inferior and middle frontal gyrus during emotion regulation and inhibitory control predicted individual stress burden during COVID-19 (14). Furthermore, acute and chronic stress has negative effects on reward sensitivity, the tendency and reactivity of individuals to approach and respond to reward-related stimuli which is an important mechanism in healthy adaptation under stressful circumstances. Activity in the mesostriatal reward regions is associated with reward-related processing and positive experience (15) and could be a potential biomarker of stress resilience (16).

#### Personal aspects of coping

Personality, a bundle of interrelated behavioural, cognitive, and emotional patterns, can both facilitate and impair coping. Assuming a five factor structure of personality, higher levels on the dimensions extraversion, conscientiousness, and openness have been linked to more Engagement coping (17), more problem-focused coping as well as less emotion-focused coping (18). Disengagement coping has been associated with higher levels of neuroticism and lower levels of optimism, conscientiousness, and agreeableness (17). Moreover, personality traits have been connected to specific coping strategies. For instance, problem-solving and cognitive restructuring have been positively linked to extraversion, conscientiousness, and negatively to neuroticism. Neuroticism also predicted problematic strategies like wishful thinking, withdrawal, and emotion-focused coping. Support seeking was associated with neuroticism and extraversion (19). Social aversive personality traits, as described by the Dark Triad (20), have been reported in connection to coping: machiavellianism and psychopathy in a negative relation, narcissism in a positive relation to problem-focused coping (21).

Furthermore, a bidirectional link between psychopathology and coping has been established. Greater psychopathological symptom load has been associated with the domain of maladaptive coping (e.g., suppression or avoidance), Disengagement coping, and the strategies emotional suppression, avoidance, and denial (22) and self-blame, rumination, and catastrophizing (23). More specifically, maladaptive coping (i.e., avoidance and denial) has been negatively linked to depression (24), more so than the positive association with adaptive coping (8). During the early stages of the COVID-19 pandemic, avoidance-based coping strategies were linked to increased psychological distress, whereas approach-oriented strategies—especially positive reframing—were associated with lower levels of depression and anxiety (25). Additionally, maladaptive personality traits were found to contribute to psychological distress during COVID-19, moderated by maladaptive, avoidance-oriented coping strategies (26).

#### Social aspects of coping

Coping, however, does not take place in a social vacuum. The impact of social networks on emotional regulation, decision-making, and resilience has been demonstrated to be substantial as social networks can directly reduce stress and promote adaptive coping strategies through support (27). Social support refers to the emotional (i.e., receiving empathy, validation or encouragement), informational (i.e., getting advice, guidance or feedback), or practical assistance (e.g., financial assistance, help with tasks, or providing resources) received from others (28) and promotes psychological adjustment to stressful conditions (29). A bidirectional association of social support with better mental and physical health has been demonstrated (30) and stable social networks have been shown to act as a buffer against stress and to encourage healthy coping behaviours (31). During the COVID-19 pandemic, in which social interactions have been drastically altered, emotional social support was negatively associated with perceived psychological distress (32). Moreover, individuals tend to look to others for guidance when in doubt about how to behave in uncertain situations (33). It is especially strong in situations that are ambiguous, new, or crisis-like, where the correct course of action is unclear, as happening during the COVID-19 pandemic. However, instrumental support seeking (e.g., getting advice from others about how to behave) may heighten awareness of one’s limited control over situational factors related to the pandemic and has been associated with increased psychological distress (32).

#### Historical impact

Moreover, ways of coping are likely to be learned from others, such as parents or peers, and may therefore show stability across generations and social contexts. Within a life-span, individuals exhibit changes in their preferred coping measures, with a tendency towards secondary control strategies in the second half of life (34). Whilst a normative decline in situational coping in older age has been reported (35), challenging life events have been ascribed as having training effect, leading to the development of better coping strategies over time (36, 37). Choi et al. (38) showed that women who exhibited pre-pandemic psychological resilience to lifetime trauma, had higher well-being and significantly lower depression, anxiety, and PTSD symptoms during COVID-19. Therefore, dealing with highly stressful situations cannot be studied in isolation, as the impact of life events and their accumulation on an individual’s health and well-being depends on (changing) socio-political as well as personal conditions, such as personality traits, neurobehavioural regulatory skills, and perceived social support.

These individual changes in times of crisis can be described by the accentuation hypothesis (39). This model assumes that individual differences in personality facets are magnified when people experience profound discontinuities: Ambiguous situations press individuals to react with little or no information available about how to behave adaptively so they rely more heavily on their habitual practices, meaning that rank positions within a distribution remain stable whilst distances between individuals increase. These increments are likely to be revealed during transitions into unpredictable new situations, as occurring on a macro-level during COVID-19.

### Coping with COVID-19

The COVID-19 pandemic brought about a large number of ambiguities, for example a considerable change in daily life, disruptions of usual routines, worries about becoming unemployed, an omnipresent threat of one’s own health and major concerns about infection and potential death, grief and loss of loved ones, and great uncertainty about how to behave with others (40, 41). In response to the large-scale threat of the coronavirus, many countries have imposed extensive restrictions to limit the number of infections within the population that have been associated with an increase in mental health problems (42).

The magnitude of the COVID-19 pandemic is unprecedented in recent human history, leading to an increased need for coping with all sorts of stressors. However, many coping resources, such as social support, have been dramatically limited due to governmental restrictions, posing an exceptional challenge to an individual’s regulatory capacities. Earlier infectious disease outbreaks have been subject to coping research. In a narrative synthesis of coping with previous infectious diseases in order to provide practical considerations in the beginning of the COVID-19 pandemic, several coping responses were reported as favourable, such as problem-focused coping (e.g., seeking out alternative healthcare services, taking infection control measures, monitoring of information pertaining to the outbreak), seeking social support, distraction and mental avoidance, and positive appraisal of the situation (e.g., adopting a positive attitude, placing confidence in the government’s ability to manage the situation or their healthcare system) (40). For coping with the COVID-19 pandemic, positive thinking, active stress coping, and social support were found to possess protective qualities of psychological well-being in Austrians (43), distraction, active coping, and seeking emotional support in Americans (44), and acceptance, humour, and planning in Greek individuals (45).

When investigating coping profiles instead of specific coping strategies in the context of COVID-19, Kavčič et al. (46) found people exhibiting engaged profiles (active coping, planning, acceptance, positive reframing) to report highest levels of well-being as compared to disengaged profiles (low problem-focused coping, social support, acceptance, positive reframing), and the avoidant profile (substance use, self-blame, humour). Similarly, Kenntemich et al. (47) used the Brief-COPE inventory (48) to identify latent coping profiles during the COVID-19 pandemic in Germany and their associations with well-being. They found five profiles with different types and numbers of coping strategies: high functional, moderate functional, high functional and religious, low functional, and moderate functional and dysfunctional coping. Highly functional coping profiles were associated with greater well-being.

Health behaviour (e.g., exercise, drinking, smoking, diet) is a critical aspect of physical and mental health and well-being, and can be seen as an expression of coping efforts as individuals’ health behaviours have been shown to be significantly influenced by their stress levels. Different facets of health behaviours are then utilised as a means of coping with or managing the distress experienced in the face of adversity. This is particularly relevant in the context of the COVID-19 pandemic and its pervasive threat to personal health. Significant changes in health behaviours have been reported, often with an exacerbation of health-harming behaviours such as physical inactivity and sedentary behaviour (49–51) and increased intake of processed food (52), junk food (53), and sweets (54), and frequent unhealthy snacking (55), particularly with the purpose of coping with increased anxiety levels (56–58). Participants who reported increases in emotional eating also reported decreases in social connection (55). Similarly, more heavy cigarette smoking, more heavy alcohol consumption, and an increased screen time has been reported (59). For some individuals, however, health-related behaviours improved during the COVID-19 pandemic and led to more homemade meals (60, 61) and increased intake of fruits and vegetables (62, 63) as well as a reduction in alcohol consumption (63).

Previous research has shown changes in individuals’ health behaviour during the COVID-19 pandemic, with both improvements and deterioration, indicating an accentuation of health-related behaviours that could be a potential outlet for individual efforts to cope with changes to daily life due to COVID-19. In line with this reasoning, Acuff et al. (64) found no averaged increase in alcohol consumption across 58 countries, but 23% of increases and 23% of decreases and suggest drinking to cope with negative affect to be a potential mediator (65).

In sum, we plan to analyse a wide array of factors comprising biological, psychological, and social aspects that impact coping in a life-long process. Hence, it is important to investigate the coping efforts employed in the context of the large-scale crisis posed by the COVID-19 pandemic in consideration of pre-existing as well as concurrent conditions.

### Objectives

Due to the uniqueness of the Rostock Longitudinal Study (“ROstocker LängsschnittStudie,” ROLS) in terms of its duration, research focus, and the lack of comparable studies, many of the investigations presented here are exploratory in nature. Our work will start with longitudinal analysis of data on coping. (1) Using data from previous measuring points, we will identify qualitatively distinct coping profiles across three decades of adulthood and examine probabilistic transitions between these profiles over time. Moreover, we will evaluate whether certain profiles are associated with more favourable or adverse mental health and well-being outcomes in late middle age. (2) We will assess the role of these lifelong coping profiles in buffering or facilitating the effects of COVID-19-related restrictions, exploring whether certain profiles are linked to more or less psychopathological strain. (3) Similarly, we plan to characterise longitudinal trajectories of alcohol use to cope, examining differences in intercepts and slopes, and investigate whether trajectory membership of drinking alcohol to cope motives predicts actual alcohol consumption during the COVID-19 pandemic. To extend this developmental coping perspective, we will examine neurobehavioural and social factors that may shape individual differences in stress regulation and health-related coping during a prolonged public health crisis. (4) Specifically, we will investigate the neurobehavioural basis of stress regulation during the COVID-19 pandemic by testing whether ventromedial prefrontal control and mesostriatal reward anticipation are associated with psychopathological COVID-19 strain over time, and whether these associations are mediated by self-reported control strategies. (5) We will examine the role of social integration in coping with COVID-19 by analysing egocentric social networks to assess how network structure and behavioural composition relate to health-related coping and preventive behaviours during the pandemic. (6) Lastly, the accentuation hypothesis will be tested by examining change in variability in outcomes, assessing stability in individuals’ rank positions over time, and analysing the direction and magnitude of individual changes. Shifts towards distributional extremes versus the centre will distinguish accentuation from regression to the mean and will be modelled using regression analyses with coping trajectories and multidomain predictors.

In line with a biopsychosocial framework, neurobehavioural factors are conceptualised as biological markers of individual differences in stress regulation that shape psychological coping processes and their effectiveness within social contexts, without assuming direct or deterministic effects. Together with longitudinal coping profiles and egocentric social network characteristics, this approach allows investigation of how biological, psychological, and social factors jointly contribute to resilience and vulnerability during a prolonged public health crisis.

## Methods and analysis

### Study setting and design

**Figure 2:**
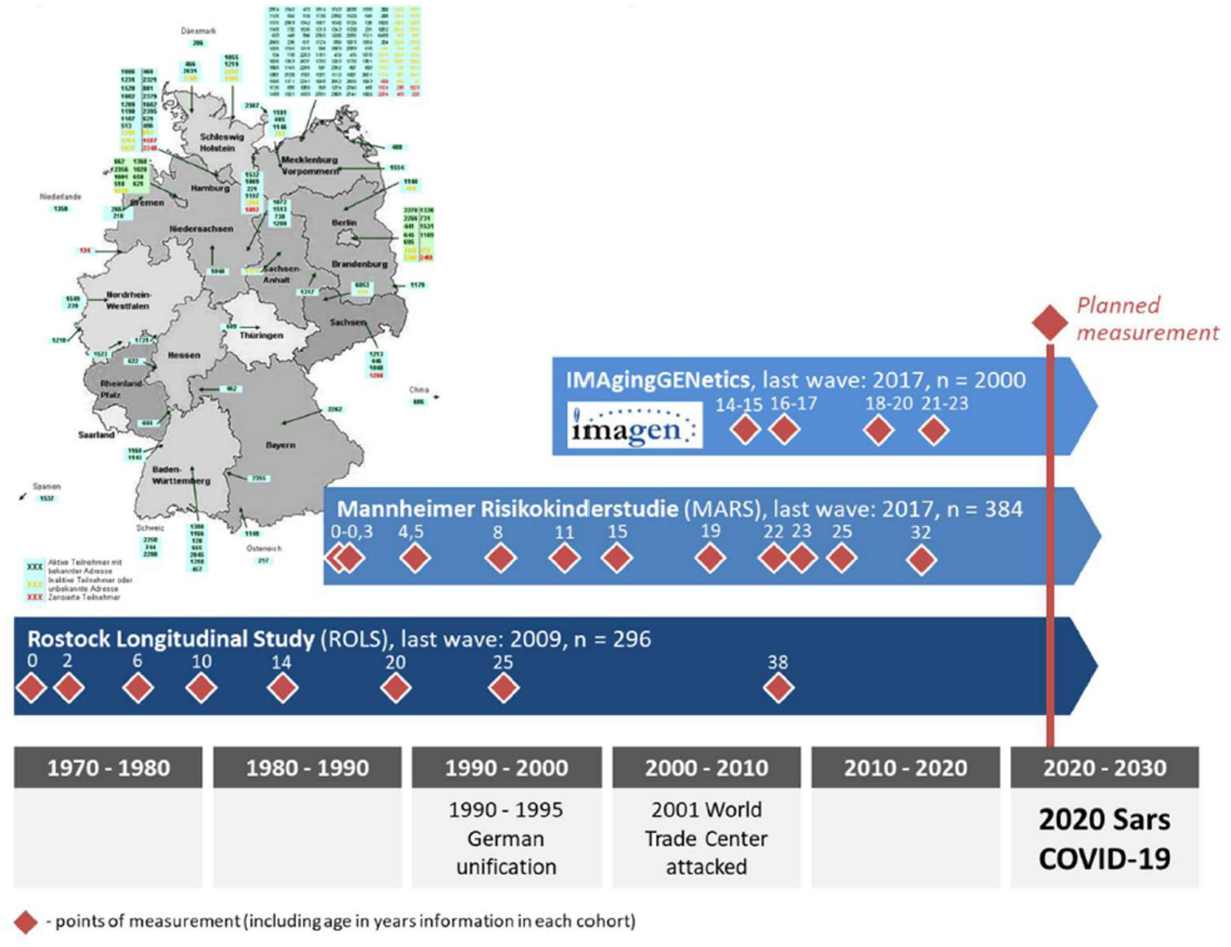
CoviDrug consortium.

The primary design of the ROLS is a single-cohort, long-term longitudinal study in a single centre setting. It is a prospective, mixed-methods observational study with both quantitative and qualitative data currently undergoing its 9th wave of data collection. The study began in 1970 and was followed by measuring time points in 1972, 1976, 1980, 1984, 1990, 1995, and 2008 (Figure *2*, dark blue beam). The ROLS is traditionally investigating how individuals cope with far-reaching socio-political changes, e.g., the German Unification in 1990. Now entering its 9^th^ wave, it aims to shed light into the interplay of long-term and short-term conditions of coping with COVID-19 in Germany, investigating the effects of the pandemic on physical and mental health as well as health behaviour whilst taking into account existing individual and contextual factors. The 9^th^ wave of ROLS is part of the multicentre consortium CoviDrug *“The role of pandemic and individual vulnerability in longitudinal cohorts across the life span: refined models of neurosociobehavioral pathways into substance (ab)use,”* funded by the German Research Foundation (Deutsche Forschungsgemeinschaft, DFG). The overarching aim of the consortium is to examine how pandemic-related stressors interact with individual vulnerability factors across the life span to influence substance use and related outcomes and includes the fourth follow-up of the Mannheim cohort of the imaging and genetic study *IMAGEN* (66) as well as the prospective study *Mannheimer Risikokinderstudie* (Manheim Study of Children at Risk—MARS, light blue beams in Figure 2). Each cohort operates within its own primary research focus and conceptual framework, reflecting differences in original study aims and disciplinary perspectives. To address a shared consortium research aim, the cohorts aligned their assessment strategies by harmonising key constructs whilst retaining cohort-specific theoretical frameworks. The present protocol reports only the aims and planned analyses of the current study and was prepared in accordance with the updated SPIRIT reporting guidelines for protocols of clinical trials (67, 75).

### Selection of subjects

Aside from sample attrition, the original study sample of 296 individuals (148 female, 148 male) has not changed throughout the years. If participants discontinued the study, they were not replaced via additional recruitment. Inclusion of the original sample was part of a neonatology project of the former East Germany (DDR), in which all deliveries in two hospitals in Rostock, Germany were examined between May 1970 and April 1971 (68). 150 new-borns were deemed at risk by paediatricians, due to either complications during the pregnancy (e.g., gestosis, liver or kidney disease of the mother) or delivery (e.g., bradytocia, prolapse of the umbilical cord, pelvic presentation of the foetus), 150 sex-equalled controls were sampled from all screened new-borns. At T1 in 1972, the sample was set to 294, as six participants could not be reached in day care settings, where all infants were examined. Based on the information gathered from mothers at the time, biological and social risks were assessed in an extensive neuropsychiatric battery. In later years, it turned out that the early risk assessments made by neonatologists at birth were not predictive, neither for physical nor for psychological development. Therefore, the ROLS should be regarded as a normative sample as opposed to a risk sample. However, the accumulation of risks in childhood, regardless if of biological or social nature, turned out to be predictive in many ways (69).

### Sample size

The study is embedded in a closed longitudinal cohort that has been followed for over 50 years. Consequently, the expected sample size is determined by cohort availability and participants’ willingness to take part at the time of data collection, rather than by recruitment targets. The data collection phase of the 8th wave took place between 2006 and 2009. Out of originally 294 mother–child pairs, 211 participants took part (71% retention rate after almost 40 years). Given the long follow-up interval, expected age-related attrition, and loss to follow-up typical of long-term cohort studies, a reduction in participation is anticipated. In addition, the current wave includes assessments related to COVID-19–related experiences, which represent a potentially sensitive topic and may further affect participation rates. Based on these considerations and prior participation patterns, we expect to recruit approximately 150 participants in the current wave (~50% retention rate after 55 years). All eligible participants will be systematically contacted and invited to participate using repeated and respectful procedures. At the beginning of recruitment in February 2022, participants were around 52 years old. Data collection started in April 2022. At the time of submission of the study protocol, data of 103 individuals have been collected. We expect a subsample size of 50% for blood sampling and imaging procedures as they are unfamiliar to our participants and may be met with hesitation.

### Constructs

Measures consist of extensive online questionnaire packages, a semi-structured face-to-face (F2F) interview including mapping the social network (via the “Network Canvas”-app), blood sampling for genetic analysis, functional magnetic resonance imaging (fMRI), ecological momentary assessment (EMA) using smartphones, and an online follow-up survey 14 days post interview. The assessment battery was harmonised across the three cohorts and not all collected measures are analysed in the present manuscript. An in-depth description of all measures can be found in the Supplementary material.

### Recruitment and participant timeline

Participants are sent an invitation letter and detailed information about the 9th wave of the ROLS by mail and asked to send back their signed informed consent prior to providing any study data. Additional consent forms are included for optional study parts (blood sample, fMRI), and upon return, screened for potential contraindication (e.g., pacemaker). Non-responsive participants are then contacted by phone, in case of a valid phone number, receive reminder letters or are visited at home by a member of the team to see if their address is still correct. As Germany does not have a central registry for its citizens, identifying the participants’ whereabouts is a time- und resource-consuming task. As participants have indicated their consent to be contacted for future surveys during previous waves, inquiries are sent to the local registry’s office to update personal data such as last name and/or address.

Participants are provided with individualised access to online questionnaires 4 weeks before the F2F interview. Completion of the questionnaire is estimated to take approximately 2 h. To reduce participant burden and fatigue, the questionnaires are divided into six sections, each requiring approximately 20 min to complete (“Alltag und Psyche”-Daily life and psyche, “Gesundheit”-health, “Stress und Stärken”-stress and strengths, “Zur Person 1”-about the person 1, “Zur Person 2”-about the person 2, “Corona und Netzwerk”-corona and network). Participants are allowed to complete the questionnaire in multiple sessions. Progress is saved automatically, allowing participants to pause and resume completion without loss of previously entered data. To ensure data completeness and standardisation, all items within each section require a response before participants can proceed. To minimise missing data and participant attrition, completion of the online questionnaires is monitored by the research team. In cases where one or more sections remain incomplete, participants will receive reminders via email and, if necessary, follow-up contact by telephone. The semi-structured interview, lasting between two and 3 h, is conducted in person in accordance with the prevailing local regulations pertaining to the COVID-19 virus until such time as they were rescinded by the German government in March 2023. For participants who can or will not come to the clinic, home visits are rendered possible throughout Germany and neighbouring countries. The mapping of the social network is collected via the “Network Canvas Interviewer”-app which is installed on Android-based study tablets. The neurobehavioural battery is performed during the F2F appointment, either using a laptop in case of home visits or using functional magnetic resonance imaging in cooperation with the Centre for Diagnostic and Interventional Radiology (ZfDIR) at the University Medical Centre Rostock. An optional ecological momentary assessment (EMA) of 14 days following the F2F meeting is employed, completed by a 5-min, follow-up online questionnaire about the participant’s experiences with the smartphone assessment as well as additional scales. Participants receive an expense allowance depending on their participation amounting up to 150€ (questionnaires and F2F interview–50€, fMRI–50€, EMA–50€).

### Recruitment and sampling procedure

Research assistants are trained to promote quality assurance and will attend regular meetings to explore any difficulties encountered. The training encompasses an explanation of the rationale behind the study, how to take informed consent, how to administer online questionnaires, how to conduct the semi-structured interview and observations in a standardised procedure. Blood samples are taken and fMRI is conducted by trained medical staff of the clinic in cooperation with radiological technologists. Any unexpected, adverse events encountered during F2F-meetings will be reported and assessed within the study team.

### Plans for assessment and collection of outcomes

Participants who have given their informed consent are contacted individually by phone to arrange an appointment for the F2F meeting. Applying a two-stage process, participants receive an e-mail containing links to the online questionnaires, which are to be filled in before meeting with a researcher in person. Preliminary analyses of questionnaire data are used to feed the semi-structured interview. Interviews are not recorded but instead notes are taken by the research assistant. As part of this F2F interview, the social network will be mapped, the blood sample is taken, and the neurobehavioural battery is conducted.

### Data management

Personal information on potential and enrolled participants are maintained in order to protect confidentiality by replacing personal data with a pseudonym throughout the study and its use within the consortium. Electronic data such as online questionnaires, fMRI, and social network data will be stored on servers of the University Medical Centre Rostock, with only the research team having access to study and personal data. Paper-and-pencil data will be stored in the ROLS archive in fireproof lockers. Blood samples are being stored at a lab specialised in biological research data (Biobank Psychischer Erkrankungen, Zentralinstitut für Seelische Erkrankungen, Mannheim, Germany).

### Plans for collection, laboratory evaluation, and storage of biological specimens for genetic analysis in this trial

Pseudonymised blood samples for genetic analysis are stored at a cooperating institute at the Central Institute of Mental Health in Mannheim. DNA will be extracted and examined for the existence of risk genes of substance use using genome-wide association studies. Participants indicate their consent for future use in ancillary studies; if not their blood sample is destroyed after the current investigation. The fMRI data are not screened by radiologists but participants have access to their data on request. On completion of the study, the data will be analysed and a final study report will be written. Participants will be notified of any publications if they have expressed an interest. Participants have the opportunity to review all their data and the right to have their data deleted and blood sample destroyed.

### Data analysis

Data will be analysed in two ways simultaneously. First, ROLS data will be entered into combined analyses of all consortional data. The foundations for this comparative work have been laid, including the development of tools for harmonising all three data sets, notably a Natural Language Processing-based semantic search approach using embedding models to identify semantically similar questionnaire items across heterogeneous instruments, thereby reducing manual effort and supporting valid cross-study harmonisation (70).

Second, ROLS data will be analysed within the study’s framework. Analyses of this kind are limited by the sample size, however, they will benefit from the long period analysed prospectively, the number of measurements, and the richness of the data. Letter points will allow for effective imputation methods, since certain constructs were investigated on multiple occasions. In the context of secondary data analysis, the basic principle is to “make the best of what you have” (71), meaning, that—to certain extent—methodologies must be adapted to the specific characteristics of the data being analysed.

Data of the 9th wave will be analysed once participants have completed all study parts and no interim analysis will be performed. The data collection process will be terminated, when all participants expressing interest in participation are met in person. As for the analysis of the hypotheses mentioned above, methods will be the following: (1) Coping profiles and their trajectory across adulthood will be analysed using latent profile transition analysis implemented as a hidden Markov model. Habitual coping, assessed consistently with the Stress Management Questionnaire (72) at three time points (6th, 8th, and 9th waves), will be used to identify qualitatively distinct profiles and probabilistic transitions over three decades, whilst accounting for measurement error. This person-centred approach is appropriate considering the theoretical interest in stable and changing patterns of coping rather than continuous change in individual strategies. Given three waves over decades, we will use Full Information Maximum Likelihood (FIML) to handle missing data. Post-hoc profiling of coping trajectories and their associations with health and well-being in late middle age will include analyses of variance and appropriate non-parametric alternatives. (2) The role of lifelong coping profiles during the COVID-19 pandemic will be investigated using linear mixed-effects models (LMMs) with monthly psychosocial strain ratings (on a Likert-type scale ranging from 1 to 5) over 3 years of the pandemic (March 2020 to March 2023) as the outcome. To address potential memory bias during retrospective data collection, participants are given brief descriptions of currently valid COVID-19 restrictions for each month. Coping profile membership will be included as a fixed effect, with piecewise time segments representing stricter versus less restrictive pandemic periods. Random intercepts and random slopes for time will model baseline differences and individual variation in trajectories. To account for varying recall periods between the month being rated and survey completion, recall delay (in weeks) will be included as a covariate in all LMMs. Interactions between coping profiles and piecewise time will test whether certain profiles buffer or exacerbate psychosocial strain. (3) Alcohol use to cope trajectories from emerging adulthood to late middle age will be analysed using latent growth mixture modelling on the alcohol-related items of the subscale substance use to cope (Stress Management Questionnaire) for three time points (6th, 8th, and 9th waves), missing data will be handled using FIML. Trajectory membership will be used to predict alcohol consumption during the COVID-19 pandemic by fitting linear regression models, and potential sex effects will be explored using the three step approach with multinomial logistic regression models. Whilst the identification of coping and alcohol-use trajectories across adulthood is primarily exploratory given the uniqueness of the cohort and the absence of comparable long-term studies (Objectives 1–3), the investigation of neurobehavioural and social influences on coping during the COVID-19 pandemic (Objectives 4–6) is theory-guided and builds on prior evidence regarding stress regulation, executive control, reward processing, and social integration. (4) Functional MRI data will be analysed using standard pipelines in SPM, running in MATLAB, including standard preprocessing. For task-based fMRI, individual-level contrasts for the onset of event types will be modelled and general linear models (GLMs) will be specified for the Stop Signal Task (Go, Successful Stop, Failed Stop) and Monetary Incentive Delay task (cue, anticipation, outcome), with regressors convolved with the canonical hemodynamic response function. At the group level, we will extract activation measures from regions of interest, including ventromedial prefrontal cortex (vmPFC) for cognitive control and mesostriatal regions for reward anticipation that will first be examined as predictors of longitudinal psychopathological strain during the COVID-19 pandemic using linear mixed-effects models (LMMs). Monthly strain ratings will serve as the outcome, with ventromedial prefrontal cortex activation (Stop Signal Task) and mesostriatal reward anticipation (Monetary Incentive Delay task) included as time-invariant predictors. Piecewise time segments representing periods of stricter versus less restrictive measures will be specified, along with random intercepts and random slopes for time, to model individual differences in baseline strain and trajectories. Interactions between neural activation and time will be tested to assess buffering or vulnerability effects across pandemic phases. In a second step, self-reported control strategies as assessed with the Optimisation in Primary and Secondary Control Scale (73) during the pandemic will be examined as potential mediators of the association between neural activation and psychopathological strain. Mediation analyses will be conducted using multilevel mediation models, with neural activation predicting control strategies and control strategies predicting longitudinal strain, whilst accounting for the repeated-measures structure of the outcome. Indirect effects will be estimated using appropriate methods for multilevel data (e.g., Monte Carlo or bootstrapped confidence intervals), allowing evaluation of whether neurobehavioural markers influence pandemic-related strain through engagement in specific coping strategies. MPRAGE, resting state, and DTI data will be analysed by the consortium later on. (5) Social network analysis will consist of an examination of key egocentric metrics—including network size, density, degree centrality, and constraint—that will be calculated and visualised using R with the *igraph* and *egor* packages, or Network Canvas’s built-in analytics. Alter-level data on vaccination behaviour, adherence to COVID-19 restrictions, and health behaviours relevant to coping (e.g., physical activity, alcohol consumption) will be used to characterise the behavioural composition of each ego network. In a second step, Exponential Random Graph Models (ERGMs) for small networks (*ergmito*) (74) will be fitted to investigate homophily in vaccination and clustering in preventive and health-related behaviour during the COVID-19 pandemic, using exact statistical calculations instead of simulation-based approximations. Separate models will be specified for vaccination and restriction adherence as well as for health behaviours conceptualised as coping efforts (e.g., exercise, alcohol use). These analyses will test whether similarity in health-related coping behaviours is greater than expected by chance and whether socially embedded behavioural patterns contribute to adaptive or maladaptive coping during a health crisis. (6) For the accentuation hypothesis, several ways will be tested to verify it. First, deviations in panel samples should increase for several outcomes, such as alcohol consumption or positive health behaviour. If deviations increase within the health behaviour data, the hypothesis should be tested that persons remain stable with regard to their ranks in outcomes before and during or after the crisis. In a third approach, individual changes in different outcome measures should be analysed for their direction and magnitude. Changes towards the edges of distributions will speak for the accentuation hypothesis, changes towards the centre for a regression to the mean. Changes of both kinds will then be modelled as the dependent variable in several regression models including coping trajectories mentioned above and predictors from various domains, such as social relations, COVID-associated burdens, brain associated mechanisms or genetic risks (PRS). Analyses were restricted to measures relevant to the predefined research questions of the ROLS and other harmonised measures are reserved for future consortium analyses.

Together, these analyses integrate developmental coping profiles, neurobehavioural markers, and egocentric social network characteristics to identify individual and social determinants of health behaviours and psychosocial strain during the COVID-19 pandemic, informing public health strategies for promoting adaptive coping and preventive behaviour in future health crises. As this study represents a long-term, evolving cohort project, additional opportunities for exploratory analyses may arise as new questions, methods, and standards emerge. These investigations will be clearly distinguished from the pre-specified, theory-driven objectives (Objectives 4–6) and the primary exploratory objectives (Objectives 1–3), and will be undertaken as hypothesis-generating analyses when scientifically justified, for example using emerging methods such as machine learning.

### Methods to handle protocol non-adherence and any statistical methods to handle missing data

Data will be examined to identify any patterns in the missing data and, if appropriate, use imputation, the exact method depending on the nature of missing data (e.g., simple mean imputation, multivariate imputation by chained equations, FIML). If any instructions are provided in the questionnaire’s manual for individual missing values, they will be followed.

### Plans to give access to the full protocol, participant level-data and statistical code

Standard operating procedures, participant level-data as well as code for statistical analysis will be shared within the consortium. The data collected as part of this study are intended to be made available for scientific access in the future and to serve as a foundation for subsequent collaborative research. Whilst the current protocol focuses on cohort-specific aims and analyses, the scope of data collection exceeds the analyses presented here, as the present research team does not currently have the capacity or expertise to conduct all potential analyses. These data will therefore support future collaborations and consortium-level investigations.

## Discussion

Understanding why individuals respond so differently to public-health crises is essential for anticipating population needs and designing effective interventions. By drawing on more than five decades of prospective data, this study provides a rare opportunity to examine how enduring coping profiles - shaped by interconnected biological, psychological, and social influences - affect adaptation to the unprecedented challenges of the COVID-19 pandemic. Identifying stable coping profiles and their long-term determinants can clarify mechanisms underlying resilience and vulnerability, offering insight into why some individuals maintain health-protective behaviours whilst others engage in maladaptive responses during crises. The life-course perspective applied here extends existing public-health research by moving beyond short-term or cross-sectional assessments, thereby enabling more precise prediction of crisis responses. Furthermore, comparing findings across national cohorts within the CoviDrug consortium increases the potential for generalizable conclusions relevant to future pandemics or other large-scale stressors. Ultimately, this work may support the development of targeted, evidence-based prevention strategies aimed at fostering adaptive coping early in life, strengthening psychosocial resources, and reducing the burden of mental and physical health problems during times of societal disruption.
