# Supplementary material for: Life-long coping patterns and bio-psycho-social predictors of individual responses to the COVID-19 pandemic—protocol of the Rostock Longitudinal Study (ninth wave)
Source: Front Public Health. 2026 Mar 2;14:1753885. doi: 10.3389/fpubh.2026.1753885 (PMC12990129; doi:10.3389/fpubh.2026.1753885)
Supplement: Supplementary file 1 [file Table_1.docx]

## Description of all measures

### Sociodemographic and biographic information

Sociodemographic information is collected during the F2F-interview in accordance with the recommendations by the German Federal Statistical Office (DESTATIS), including age, gender identity, nationality, nationality of parents, marital status, household characteristics (e.g., living space, number of rooms, rent), religious denomination, education, current employment and occupation, income, financial situation, and political orientation. Life events are assessed using a self-developed list of 40 major life events (e.g., severe illness, separation/divorce, death of a loved one, lawsuit, financial struggles) occurring since the previous wave of collection with a *yes*/*no* response format as part of the online questionnaires. During the F2F-interview, for each life event subjective burden on a five-point scale (1 = *not at all* to 5 = *extreme*) as well as COVID-19 attribution (*yes*/*no*) is enquired.

### Personality questionnaires

The NEO-FFI-60 (McCrae & Costa, 2004) is a multidimensional personality inventory investigating personality on five subscales (openness, conscientiousness, extraversion, agreeableness, and neuroticism) using 60 items on a five-point Likert scale. The Substance Use Risk Profile Scale (SURPS) (Woicik et al., 2009) assesses four personality dimensions that pose a risk for substance abuse (hopelessness, anxiety sensitivity, impulsivity, and sensation seeking). It utilizes 23 items on a four-point scale (1 = *disagree*, 2 = *tend to disagree*, 3 = *tend to agree*, 4 = *agree*). The Short Dark Triad (SD3) is a 27-item self-report instrument that captures subclinical versions of narcissism, machiavellianism, and psychopathy, rated on a four-point scale (Jones & Paulhus, 2014). Strengths and Difficulties Questionnaire (SDQ) (Goodman et al., 1998) is a 25-item measure of behaviour across five domains: emotional symptoms, conduct problems, hyperactivity, peer problems, and prosocial behaviour. Initially designed for data collection in children and adolescents, it is also available for adults up to 65 years of age. Items are rated on a three-point scale and encompass a six-months-time period.

### Mental health

The Beck Depression Inventory (Beck et al., 1961; Beck et al., 1996; Hautzinger et al.) is a widely used self-report 21-item scale to assess severity of depression in adults. Individuals are asked to respond to each question based on a two-week time period. State and Trait Anxiety is assessed using the State-Trait-Anxiety Inventory (Grimm, 2009; Laux et al., 1981; Spielberger et al.) using a translated (Laux et al., 1981) short version with 10 items for each subscale rated on a seven-point scale ranging from *almost never* to *almost all the time*. Subjective stress level is assessed using the translated (Klein et al., 2016) 10-item version of the Perceived Stress Scale (Cohen et al., 1983; Klein et al., 2016). It is a five-point self-report questionnaire that evaluates the degree to which individuals believe their life has been unpredictable, uncontrollable, and overloaded during the previous month. The Satisfaction with Life Scale (SWLS) assesses global life satisfaction (Diener et al., 1985; Janke & Glöckner-Rist, 2012) on five items using a seven-point response format.

### Physical health

The Brief Symptom Inventory (BSI-18) (Derogatis, 2000; Spitzer et al., 2011) is the shortest of the multidimensional versions of the Symptom-Checklist 90-R, containing three subscales (somatization, depression, and anxiety) as well as a global scale. Responses are rated on a five-point scale, ranging from 1 (*not at all*) to 5 (*extremely*). The Zerssen list (Zerssen & Petermann, 2011) assesses health problems on two parallel forms containing 20 items each. Both versions are included, leading to 40 items in total (e.g., shortness of breath, pain in the upper or lower abdomen, vertigo, nausea, joint pain) that are rated on a four-point scale (1 = *not at all*, 4 = *extremely*). Participants have to indicate how much they have experienced each in the preceding six months as part of the online questionnaire.

The Örebro Musculoskeletal Pain Questionnaire (Schmidt et al., 2023) is implemented for assessment of physical pain. It consists of a total of 22 items related to five domains (1) pain intensity, (2) fear-avoidance beliefs, (3) depressiveness, (4) functional impairment in daily life, and (5) a list of pain location. The Fear of Pain Questionnaire (FPQ) (McNeil & Rainwater, 1998) is a self-report instrument in which 30 painful experiences are listed that are rated on a five-point scale (1 = *not at all*, 5 = *extreme*).

### Physical and mental health status

Participants are asked about their satisfaction with current mental as well health status on a five-point Kunin scale (Kunin, 1955). Furthermore, they have to indicate any personal experience with psychotherapy/ psychiatric services, previous treatment, psychiatric diagnoses, as well as psychiatric disorders that run in the family. Similarly, participants have to provide details on any chronic and hereditary illnesses as well as information on their height and weight which is used to calculate their Body Mass Index (BMI). They are also asked about their COVID-19 vaccination status, their reasons for/against the vaccination, as well as severity and symptom load of any previous infections with the coronavirus.

### Health behaviour

The Alcohol Disorder Identification Test (AUDIT) is a simple self-report method of screening for excessive drinking and to assist in brief assessment (Babor et al., 2001; Dybek et al., 2006). It contains 10 items asking for frequency and quantity of alcohol use, as well as negative consequences due to consuming alcohol. The Severity of Dependence Scale (SDS) (Gossop et al., 1995) is a short five-item scale to measure the degree of dependence and is available for a wide range of substances. The original items were translated to German and adapted to alcohol (Steiner et al., 2008). Example items are “do you think your alcohol use is out of control?” and “did the prospect of missing a drink make you anxious or worried?”. It is rated on a four-point scale. The Drinking Motives Questionnaire (DMQ) is a widely used tool to assess drinking motives (Cooper; Kuntsche et al., 2006). Originally designed for adolescents, it has been validated for older adults (Gilson et al., 2013). In its short version, it includes 12 items on three subscales (social, coping, enhancement). Timeline Followback (TLFB) is a calendar method to obtain a quantitative estimate of an individual’s daily drinking over a given period of time (Sobell & Sobell, 1992). It is administered by the researcher during the F2F interview, as assistance by an interviewer ensures higher quality data, and encompasses the 30 days prior to the interview date. Drug-use consumption variables for different substances (e.g., cocaine, heroin, amphetamines) are included as well.

The Fagerström Test of Cigarette Dependence (Fagerström, 2012; Heatherton et al., 1991) measures nicotine dependence on six items, that are summed up to form a global score. It is incorporated into the F2F interview, only for participants who reported currently smoking on a regular basis. A German translation is openly accessible by the German Cancer Research Centre. Furthermore, participants are asked about their duration of smoking, if they had any breaks since they first began, and any changes in smoking habits since start of COVID-19 pandemic.

Three Factor Eating Questionnaire R-18 (Karlsson et al., 2000) is a revised version of the Three Factor Eating Questionnaire by Stunkard and Messick (1985) and assesses eating patterns on 18 items, three subscales (cognitive restraint, uncontrolled eating, emotional eating). An excerpt regarding eating habits consisting of 11 items that are rated on a five-point scale is taken from the Fragebogen zur Erfassung des Gesundheitsverhaltens (Questionnaire to assess health behaviour) (Dlugosch & Krieger, 1995). Example items are „I often eat something in between meals.”, “I usually prepare my own meals.”, “I often eat something just before going to bed.”.

Moreover, participants are asked about the quantity of food intake, their water intake, quantity of meals per day including in-between meals and snacks, if they are currently following a particular diet (e.g., vegetarian, vegan, paleo, low-carb, etc.), have any desire to make changes regarding eating behaviours, and have made any changes to their eating behaviours since the beginning of the COVID-19 pandemic.

The Pittsburgh Sleep Quality Index Questionnaire (PSQI) (Buysse et al., 1989; Riemann & Backhaus J, 1996) is one of the most widely used tools to assess general sleep quality as it is fairly simple, validated, reasonably quick, and easy to administrate. It measures subjective sleep quality, sleep latency, sleep duration, habitual sleep efficiency, sleep disturbance, use of sleeping medication, and daytime dysfunction on 19 items. A global score of overall sleep quality can be calculated by adding up the single scores of these dimensions. Most items are incorporated into the F2F interview, except for the sleep disturbance subscale that was part of the online questionnaires.

Godin-Shephard Leisure-Time Physical Activity Questionnaire is a common self-report measure of physical activity using four items with the first three question seeking information on the number of times one engages in mild (e.g., yoga, golf, easy walking), moderate (e.g., fast walking, tennis, easy bicycling), and strenuous (e.g., running, vigorous long distance bicycling, vigorous swimming) leisure-time physical activity (Godin, 2011; Godin & Shephard, 1985). To obtain a total score, each session is multiplied by 3, 5 or 9 metabolic equivalents (METs) and summed up. Furthermore, participants are asked about their preferred physical activities as well as its weekly quantity and duration during the F2F-interview as well as an estimate of their usual step count and their reasoning behind their exercise behaviour.

Participants are asked how important certain aspects of their health behaviour are to them on four items each (e.g., “*X* is extremely important.”, “I think people who don't make an effort to *X* are quite irresponsible.”, “*X* is of great value in life.”, “I think everyone should endeavour to *X*.”). X being a) a good night’s sleep, b) a balanced diet, c) exercising, d) nicotine and e) alcohol abstinence. Health importance is assessed twice, incorporated into the online questionnaires regarding participants’ current attitudes as well as during the F2F-interview regarding their attitudes in the years leading up to the pandemic to allow for comparison between time points.

The Mindfulness Attention Awareness Scale (Brown & Ryan, 2003; Michalak et al., 2008) is used to measure (self-assessed) dispositional mindfulness comprising 15 items on a 6-point Likert scale (Likert) ranging from 1 (*almost never*) to 6 (*almost always*). During the F2F interview, participants are asked if they consciously take breaks in everyday life, practice any form of structured mindfulness (e.g., Yoga, meditation, Tai Chi, Qi Gong, etc.), and if they made any changes in their mindfulness practice since start of COVID-19 pandemic.

Information on media consumption is adapted from the CoRonavIruS Health Impact Survey (CRISIS) (Nikolaidis et al., 2021) and contain the following question: how much time they spend watching television (including streaming services), on social media (e.g., Facebook, Instagram, TikTok, as well as Messenger services such as WhatsApp), playing video games, as well as any changes in media consumption since start of COVID-19 pandemic.

Furthermore, participants are asked how satisfied they are with different aspects of their health behaviour. Example items are “How satisfied are you with your smoking habits?” or “How satisfied are you with your alcohol use?” rated on a five-point Kunin scale (1 = *dissatisfied*, 2 = *rather dissatisfied*, 3 = *neutral*, 4 = *rather satisfied*, 5 = *satisfied*).

### Coping

The Stress Management Questionnaire (Erdmann et al., 1985) uses a five point scale to assess habitual coping mechanisms in individuals aged 20 to 79, comprising 19 subscales with 6 items each (e.g., need for social support, avoidance, rumination, self-blame). Responses on a five-point Likert scale are summed up to form a subscale score. The short version of the Selection Optimization and Compensation Scale (SOC (M. M. Baltes et al., 1999; P. B. Baltes & Baltes, 1989) assesses four strategies (elective selection, loss-based selection, optimization, and compensation) using twelve items. Each item contains two statements, a target SOC strategy and a non-SOC-related alternative strategy. Participants choose the strategy that best describes their usual behaviour. SOC-strategy use is indicated by the sum of selected targets. A short version of the Optimization in Primary and Secondary Control (OPS; (Heckhausen et al., 1998) is implemented, using 19 of the original 44 items on five subscales: selective primary control, compensatory primary control, selective secondary control, compensatory secondary control, as well as optimization (only three items). The participants respond to each item on a five-point Likert response scale from 1 (*not at all true of me*) to 5 (*very true of me*). Item scores are averaged to create the composite score. Higher scores indicate greater control on that subscale. Tenacious Goal Pursuit and Flexible Goal Adjustment scale (Brandtstädter & Renner, 1990) is designed to assess assimilative and accommodative strategies on a dispositional level on 30-item Likert-type scale ranging from 0 (*strongly disagree*) to 4 (*strongly agree*). After item reversals, the 15 items comprising each subscale were averaged. Higher scores indicate greater coping on that subscale.

### COVID-related questionnaires

Individual risk perception of getting infected with the COVID-19 virus is assessed using a six-item scale developed by the authors. Participants have to rate their agreement on a five-point scale (1 = *not at all*, 2 = *barely*, 3 = *moderately*, 4 = *quite a lot*, 5 = *a lot*). The following items are included: “How serious do you personally feel the COVID-19 problem is?”, “How dangerous do you personally think COVID-19 is?”, “How worried are you that you could be infected with COVID-19?”, “How worried are you that you could become seriously ill with COVID-19?”, “How likely do you think it is that you will become infected with COVID-19?”, and “How likely do you think it is that you will become seriously ill with COVID-19?”. To asses COVID-related media consumption, participants are asked how much they used different forms of media to stay updated with current COVID occurrences (e.g., television, radio, social media, podcasts, online news outlets, newspaper, internet) on a five-point scale (1 = *barely*, 2 = *a little*, 3 = *moderately*, 4 = *quite a lot*, 5 = *a lot*) at two different time points: during the first lockdown in Germany (March to May 2020) and the two weeks preceding the F2F interview. COVID-19 knowledge is determined using a short knowledge test consisting of 13 statements using a translated and adapted version of (Amram et al., 2021). Participants have to indicate if that statement is true or false. Example items of this scale are “Antibiotics can be used to treat the COVID-19 virus” and “Eating garlic can lower your chances of getting infected with the COVID-19 virus”. We added the following items: “People who are infected with the SARS-CoV-2 coronavirus but do not show any clinical symptoms cannot transmit the virus to others.”, “Being able to hold your breath for at least 10 seconds without having to cough afterwards ensures that you are not infected with the SARS-CoV-2 coronavirus.”, “Vaccines against the SARS-CoV-2 coronavirus alter DNA.”, “Influenza and pneumococcal vaccines do not protect against the SARS-CoV-2 coronavirus.”, “The most important measures to protect against infection with the SARS-CoV-2 coronavirus are vaccination, keeping your distance from other people, wearing a face mask, and washing your hands frequently with soap and water.”, and “The long-term effects of the vaccine have not been sufficiently researched.”. To understand public perceptions of government responses to COVID-19 we utilized the COVID-SCORE-10 (Lazarus et al., 2020) to asses trust in government and population risk of exposure on a 10-item scale. The original scale has been translated by bilingual researchers and translations converged*.* To assess the trajectory of individual burden of governmental restrictions in response to COVID-19 a self-developed questionnaire is used: Participants have to rate their subjective burden for each month between March 2020 and March 2023 on a five-point scale (1= *not at all* to 5 = *extreme*). To reduce memory bias, a brief description of governmental restrictions is given for each month. For every month with a burden ≥3 (= *moderate*), participants have to indicate their individual reason (e.g., loss of employment, conflicts with loved ones). An adaptation of the CoRonavIruS Health Impact Survey (CRISIS) (Nikolaidis et al., 2021) is implemented in the F2F-interview. It is covering key domains relevant to mental distress and resilience during the pandemic and is used in its follow-up version. Sections regarding health behaviour (e.g., sleep, diet, substance use) are omitted to reduce redundancy.

### Egocentric social network analysis

To collect data on social networks, a Personal Network Analysis using Network Canvas (Complex Data Collective, 2024) is employed. It provides **free and open-source** software for surveying networks and is implemented in-person and interviewer-assisted to enable the collection of higher quality data. Data on alter are collected as part of the online questionnaire to be entered into the Network Canvas Interviewer app before the F2F-interview during which more information on the relationship between the participant and her or his contacts (e.g., frequency of contact, satisfaction with relationship quality, changes since the beginning of the COVID-19 pandemic, and health behaviour such as exercise, smoking, alcohol use, diet, mindfulness) and edges between contacts are collected. The number of potential contacts is limited to ten persons per participant.

### Neurobehavioural testbattery

Stop Signal Task (SST) is a version of a classic approach to measuring response inhibition and impulse control. Participants have to respond to an arrow stimulus pointing either to the right or the left on the screen. Depending on the alignment of the arrow, the left or right arrow key has to be pressed as fast as possible. If a second arrow pointing upwards is presented right after the arrow pointing to one side, the participant has to withhold their response and not press the button. The task uses a staircase design, allowing the task to adapt to the performance of the participant.

Monetary Incentive Delay (MID) (Knutson et al., 2000) is a task to assess an individual’s reward-based learning, from reward anticipation to its delivery. Visual stimuli are utilised as incentive cues that code the magnitude of the outcomes (triangle – no reward, circle with one line – 2 points, circle with three lines – 10 points). The cue is followed by the target, a white square that appears very briefly on either the right or left side of the screen. Participants have to press the left or right arrow key while the target is displayed and receive immediate feedback on whether they have successfully obtained the reward.

Cambridge Gambling Task (CGT) (Rogers et al., 1999) is a popular neurocognitive task used to assess decision making and risk taking. Participants are presented with ten boxes at the top of a screen, each of which is red or blue in some ratio. Under one of these boxes is a yellow token, and participants must guess whether the token is under red or blue. After choosing a colour, participants can select some proportion of their allotted points to bet on their judgement.

SST, MID, and CGT are implemented using Neurobs Presentation (Neurobehavioral Systems, Inc.) and are available as both a neurobehavioural task on a laptop and during functional magnetic resonance imaging (fMRI). Additional fMRI measures include Magnetization Prepared Rapid Gradient Echo (MP-RAGE), a three-dimensional, T1-weighted gradient-echo sequence used to investigate the structural architecture of the brain, a task-free Resting state sequence measuring spontaneous low-frequency fluctuations in the BOLD signal to explore functional experience as well as Diffusion Tension Imaging for mapping the white matter of the brain.

### Ecological Momentary Assessment

Ecological Momentary Assessment (EMA) provides a method of collecting real-time data from individuals in their natural environment using mobile devices. Utilising the MovisensXS platform, participants receive questions regarding their current mental and physical state and health behaviour on their smartphone three times a day (9am, 2pm, 8pm) for a period of 14 days. For participants without a compatible phone, Nokia smartphones are provided for the duration of the survey.

An online follow-up survey is being sent via email containing a self-evaluation of the EMA. Moreover, the Hogg Eco Anxiety Scale (HEAS-13) (Heinzel et al., 2023; Hogg et al., 2021) is included, measuring anxiety relating to a multitude of ecological crises, consisting of 13 items that are rated on a four point scale ranging from 1 (*not at all*) to 4 (*almost every day*). Also included is an adapted version of the HEAS-13 regarding anxiety related to the Russian-Ukraine war as well an additional socio-political event that can be chosen by the participant.

### Genetic analyses

During the F2F appointment with the study team blood samples are taken using butterfly needles and 7.5ml EDTA tubes. Blood samples are sent to a lab for storage (Biobank Psychiatrischer Krankheiten, Central Institute of Mental Health, Mannheim, Germany) and DNA extraction. Individual DNA is investigated using genome-wide association studies (GWAS) to find genetic markers associated with substance use.

References

Amram, O., Borah, P., Kubsad, D., & McPherson, S. M. (2021). Media Exposure and Substance Use Increase during COVID-19. *International Journal of Environmental Research and Public Health*, *18*(12). https://doi.org/10.3390/ijerph18126318

Babor, T., Higgins-Biddle, J., Saunders, J., Monteiro, M., & World Health Organization (2001). AUDIT: the alcohol use disorders identification test: guidelines for use in primary health care. *World Health Organization*.

Baltes, M. M., Baltes, P. B., FR Lang, & AM Freund. (1999). *The measurement of selection, optimization, and compensation (SOC) by self report: Technical report 1999*. https://pure.mpg.de/rest/items/item_2102996/component/file_2102995/content

Baltes, P. B., & Baltes, M. M. (1989). Optimierung durch Selektion und Kompensation. Ein psychologisches Modell erfolgreichen Alterns. Advance online publication. https://doi.org/10.25656/01:14507

Beck, A. T., Ward, C. H., Mendelson, M., Mock, J., & Erbaugh, J. (1961). An inventory for measuring depression. *Archives of General Psychiatry*, *4*, 561–571. https://doi.org/10.1001/archpsyc.1961.01710120031004

Beck, A. T., Steer, R. A., & Brown, G. (1996). *Beck Depression Inventory-II (BDI-II).* https://doi.org/10.1037/t00742-000

Brandtstädter, J., & Renner, G. (1990). Tenacious goal pursuit and flexible goal adjustment: Explication and age-related analysis of assimilative and accommodative strategies of coping. *Psychology and Aging*, *5*(1), 58–67. https://doi.org/10.1037/0882-7974.5.1.58

Brown, K. W., & Ryan, R. M. (2003). *Mindfulness Attention Awareness Scale (MAAS).* https://doi.org/10.1037/t04259-000

Buysse, D. J., Reynolds, C. F., Monk, T. H., Berman, S. R., & Kupfer, D. J. (1989). The Pittsburgh Sleep Quality Index: A new instrument for psychiatric practice and research. *Psychiatry Research*, *28*(2), 193–213. https://doi.org/10.1016/0165-1781(89)90047-4

Cohen, S., Kamarck, T., & Mermelstein, R. (1983). A Global Measure of Perceived Stress. *Journal of Health and Social Behavior*, *24*(4), 385. https://doi.org/10.2307/2136404

Complex Data Collective. (2024). *Network Canvas: Interviewer* [Computer software]. Zenodo.

Cooper, M. L. Motivations for alcohol use among adolescents: Development and validation of a four-factor model. *Psychological Assessment,* 1994(6(2)).

Derogatis, L. R. (2000). *Brief Symptom Inventory-18 (BSI-18): Administration, Scoring, and Procedures*. NCS Pearson.

Diener, E., Emmons, R. A., Larsen, R. J., & Griffin, S. (1985). The Satisfaction With Life Scale. *Journal of Personality Assessment*, *49*(1), 71–75. https://doi.org/10.1207/s15327752jpa4901_13

Dlugosch, G., & Krieger, W. (1995). Fragebogen zur Erfassung des Gesundheitsverhaltens (FEG). *Swets & Zeitlinger,* 1995.

Dybek, I., Bischof, G., Grothues, J., Reinhardt, S., Meyer, C., Hapke, U., John, U., Broocks, A., Hohagen, F., & Rumpf, H.‑J. (2006). The reliability and validity of the Alcohol Use Disorders Identification Test (AUDIT) in a German general practice population sample. *Journal of Studies on Alcohol*, *67*(3), 473–481. https://doi.org/10.15288/jsa.2006.67.473

Erdmann, G., Janke, W., & Boucsein, W. (1985). *Stressverarbeitungsfragebogen (SVF)*. Verlag für Psychologie Hogrefe.

Fagerström, K. (2012). Determinants of tobacco use and renaming the FTND to the Fagerstrom Test for Cigarette Dependence. *Nicotine & Tobacco Research : Official Journal of the Society for Research on Nicotine and Tobacco*, *14*(1), 75–78. https://doi.org/10.1093/ntr/ntr137

Gilson, K.‑M., Bryant, C., Bei, B., Komiti, A., Jackson, H., & Judd, F. (2013). Validation of the Drinking Motives Questionnaire (DMQ) in older adults. *Addictive Behaviors*, *38*(5), 2196–2202. https://doi.org/10.1016/j.addbeh.2013.01.021

Godin, G. (2011). The Godin-Shephard Leisure-Time Physical Activity Questionnaire. Advance online publication. https://doi.org/10.14288/hfjc.v4i1.82 (18-22 Pages / The Health & Fitness Journal of Canada, Vol 4 No 1 (2011): Dr. Roy Shephard: A Tribute to the Dean of Exercise and Physical Activity Science).

Godin, G., & Shephard, R. J. (1985). A simple method to assesxs exercise behavior in the Community. *Canadian Journal Applied Sport Sciences,* 1985(3), pp. 141–146.

Goodman, R., Meltzer, H., & Bailey, V. (1998). The Strengths and Difficulties Questionnaire: A pilot study on the validity of the self-report version. *European Child & Adolescent Psychiatry*, *7*(3), 125–130. https://doi.org/10.1007/s007870050057

Gossop, M., Darke, S., Griffiths, P., Hando, J., Powis, B., Hall, W., & Strang, J. (1995). The Severity of Dependence Scale (SDS): Psychometric properties of the SDS in English and Australian samples of heroin, cocaine and amphetamine users. *Addiction*, *90*(5), 607–614. https://doi.org/10.1046/j.1360-0443.1995.9056072.x

Grimm, J. (2009). State-Trait-Anxiety Inventory nach Spielberger. Deutsche Lang- und Kurversion.: Methodenforum der Universität Wien. *MF-Working Paper*.

Hautzinger, M., Keller, F., & Kühner, C. Beck Depressions-Inventar (BDI-II): Überarbeitete Ausgabe. *Harcourt Test Services,* 2006.

Heatherton, T. F., Kozlowski, L. T., Frecker, R. C., & Fagerström, K. O. (1991). The Fagerström Test for Nicotine Dependence: A revision of the Fagerström Tolerance Questionnaire. *British Journal of Addiction*, *86*(9), 1119–1127. https://doi.org/10.1111/j.1360-0443.1991.tb01879.x

Heckhausen, J., Schulz, R., & Wrosch, C. (1998). *Developmental regulation in adulthood: Optimization in primary and secondary control-A multiscale questionnaire (OPS-Scales). Technical report*. https://pure.mpg.de/rest/items/item_2103212/component/file_3080635/content

Heinzel, S., Tschorn, M., Schulte-Hutner, M., Schäfer, F., Reese, G., Pohle, C., Peter, F., Neuber, M., Liu, S., Keller, J., Eichinger, M., & Bechtoldt, M. (2023). Anxiety in response to the climate and environmental crises: Validation of the Hogg Eco-Anxiety Scale in Germany. *Frontiers in Psychology*, *14*, 1239425. https://doi.org/10.3389/fpsyg.2023.1239425

Hogg, T. L., Stanley, S. K., O'Brien, L. V., Wilson, M. S., & Watsford, C. R. (2021). The Hogg Eco-Anxiety Scale: Development and validation of a multidimensional scale. *Global Environmental Change*, *71*, 102391. https://doi.org/10.1016/j.gloenvcha.2021.102391

Janke, S., & Glöckner-Rist, A. (2012). *Deutsche Version der Satisfaction with Life Scale (SWLS).* https://doi.org/10.6102/zis147

Jones, D. N., & Paulhus, D. L. (2014). Introducing the short Dark Triad (SD3): A brief measure of dark personality traits. *Assessment*, *21*(1), 28–41. https://doi.org/10.1177/1073191113514105

Karlsson, J., Persson, L. O., Sjöström, L., & Sullivan, M. (2000). Psychometric properties and factor structure of the Three-Factor Eating Questionnaire (TFEQ) in obese men and women. Results from the Swedish Obese Subjects (SOS) study. *International Journal of Obesity and Related Metabolic Disorders : Journal of the International Association for the Study of Obesity*, *24*(12), 1715–1725. https://doi.org/10.1038/sj.ijo.0801442

Klein, E. M., Brähler, E., Dreier, M., Reinecke, L., Müller, K. W., Schmutzer, G., Wölfling, K., & Beutel, M. E. (2016). The German version of the Perceived Stress Scale - psychometric characteristics in a representative German community sample. *BMC Psychiatry*, *16*, 159. https://doi.org/10.1186/s12888-016-0875-9

Knutson, B., Westdorp, A., Kaiser, E., & Hommer, D. (2000). Fmri visualization of brain activity during a monetary incentive delay task. *NeuroImage*, *12*(1), 20–27. https://doi.org/10.1006/nimg.2000.0593

Kunin, T. (1955). The Construction of a New Type of Attitude Measure 1. *Personnel Psychology*, *8*(1), 65–77. https://doi.org/10.1111/j.1744-6570.1955.tb01189.x

Kuntsche, E., Knibbe, R., Gmel, G., & Engels, R. (2006). *Drinking Motive Questionnaire (DMQ) - Revised: German, French, and Italien Versions.* https://doi.org/10.1037/t70934-000

Laux, L., Glanzmann, P., Schaffner, P., & Spielberger, C. D [Charles D.]. (1981). *Das State-Trait-Ansgtinventar (STAI): theoretische Grundlagen und Handanweisung*. Weinheim: Beltz.

Lazarus, J., Ratzan, S., Palayew, A., Billari, F. C., Binagwaho, A., Kimball, S., Larson, H. J., Melegaro, A., Rabin, K., White, T. M., & El-Mohandes, A. (2020). Covid-SCORE: A global survey to assess public perceptions of government responses to COVID-19 (COVID-SCORE-10). *PloS One*, *15*(10), e0240011. https://doi.org/10.1371/journal.pone.0240011

Likert, R. A technique for the measurement of attitudes. *Archives of Psychology,* 1932(22), pp. 5–55.

McCrae, R. R., & Costa, P. T. (2004). A contemplated revision of the NEO Five-Factor Inventory. *Personality and Individual Differences*, *36*(3), 587–596. https://doi.org/10.1016/S0191-8869(03)00118-1

McNeil, D. W., & Rainwater, A. J. (1998). Development of the Fear of Pain Questionnaire--III. *Journal of Behavioral Medicine*, *21*(4), 389–410. https://doi.org/10.1023/A:1018782831217

Michalak, J., Heidenreich, T., Ströhle, G., & Nachtigall, C. (2008). Die deutsche Version der Mindful Attention and Awareness Scale (MAAS) Psychometrische Befunde zu einem Achtsamkeitsfragebogen. *Zeitschrift Für Klinische Psychologie Und Psychotherapie*, *37*(3), 200–208. https://doi.org/10.1026/1616-3443.37.3.200

Nikolaidis, A., Paksarian, D., Alexander, L., Derosa, J., Dunn, J., Nielson, D. M., Droney, I., Kang, M., Douka, I., Bromet, E., Milham, M., Stringaris, A., & Merikangas, K. R. (2021). The Coronavirus Health and Impact Survey (CRISIS) reveals reproducible correlates of pandemic-related mood states across the Atlantic. *Scientific Reports*, *11*(1), 8139. https://doi.org/10.1038/s41598-021-87270-3

Riemann, D., & Backhaus J. (1996). *Behandlungen von Schlafstörungen: Ein psychologisches Gruppenprogramm*. Beltz.

Rogers, R. D., Everitt, B. J., Baldacchino, A., Blackshaw, A. J., Swainson, R., Wynne, K., Baker, N. B., Hunter, J., Carthy, T., Booker, E., London, M., Deakin, J. F., Sahakian, B. J., & Robbins, T. W. (1999). Dissociable deficits in the decision-making cognition of chronic amphetamine abusers, opiate abusers, patients with focal damage to prefrontal cortex, and tryptophan-depleted normal volunteers: Evidence for monoaminergic mechanisms. *Neuropsychopharmacology : Official Publication of the American College of Neuropsychopharmacology*, *20*(4), 322–339. https://doi.org/10.1016/S0893-133X(98)00091-8

Schmidt, C. O., Kohlmann, T., Pfingsten, M., Lindena, G., Marnitz, U., Pfeifer, K., & Chenot, J. F. (2023). *Verfahrensdokumentation für ÖMSPQ: Örebro Musculoskeletal Pain Questionnaire – Kurzfassung.* https://doi.org/10.23668/psycharchives.13012

Sobell, L. C., & Sobell, M. B. (1992). Timeline Follow-Back. In R. Z. Litten & J. P. Allen (Eds.), *Measuring Alcohol Consumption* (pp. 41–72). Humana Press. https://doi.org/10.1007/978-1-4612-0357-5_3

Spielberger, C. D [C. D.], Gorsuch, R. L., & Lushene, R. E. STAI Manual for the State-Trait-Anxiety Inventory. *Consulting Psychologists Press,* 1970.

Spitzer, C., Hammer, S., Löwe, B., Grabe, H. J., Barnow, S., Rose, M., Wingenfeld, K., Freyberger, H. J., & Franke, G. H. (2011). Die Kurzform des Brief Symptom Inventory (BSI -18): erste Befunde zu den psychometrischen Kennwerten der deutschen Version [The short version of the Brief Symptom Inventory (BSI -18): preliminary psychometric properties of the German translation]. *Fortschritte der Neurologie-Psychiatrie*, *79*(9), 517–523. https://doi.org/10.1055/s-0031-1281602

Steiner, S., Baumeister, S. E., & Kraus, L. (2008). Severity of Dependence Scale: Establishing a cut-off point for cannabis dependence in the German adult population. *SUCHT*, *54*(S1), 57–63. https://doi.org/10.1024/2008.07.07

Stunkard, A. J., & Messick, S. (1985). The three-factor eating questionnaire to measure dietary restraint, disinhibition and hunger. *Journal of Psychosomatic Research*, *29*(1), 71–83. https://doi.org/10.1016/0022-3999(85)90010-8

Woicik, P. A., Stewart, S. H., Pihl, R. O., & Conrod, P. J. (2009). The Substance Use Risk Profile Scale: A scale measuring traits linked to reinforcement-specific substance use profiles. *Addictive Behaviors*, *34*(12), 1042–1055. https://doi.org/10.1016/j.addbeh.2009.07.001

Zerssen, D. von, & Petermann, F. (2011). *B-LR - Beschwerden-Liste - Revidierte Fassung*. Hogrefe.
